# Risk of metabolic syndrome among law enforcement officers due to physical activity and posture behaviors

**DOI:** 10.1093/joccuh/uiad005

**Published:** 2023-11-09

**Authors:** Marquell Johnson, Saori Braun, Michelle Hecimovich, Katrina Schultz, Chantal Bauer, Anna Bohn, Jeff Janot

**Affiliations:** Department of Kinesiology, McPhee Physical Education Center 221, University of Wisconsin Eau Claire, 105 Garfield Avenue, P.O. Box 4004, Eau Claire, Wisconsin 54702, United States; Department of Kinesiology, McPhee Physical Education Center 221, University of Wisconsin Eau Claire, 105 Garfield Avenue, P.O. Box 4004, Eau Claire, Wisconsin 54702, United States; Department of Kinesiology, McPhee Physical Education Center 221, University of Wisconsin Eau Claire, 105 Garfield Avenue, P.O. Box 4004, Eau Claire, Wisconsin 54702, United States; Department of Kinesiology, McPhee Physical Education Center 221, University of Wisconsin Eau Claire, 105 Garfield Avenue, P.O. Box 4004, Eau Claire, Wisconsin 54702, United States; Department of Kinesiology, McPhee Physical Education Center 221, University of Wisconsin Eau Claire, 105 Garfield Avenue, P.O. Box 4004, Eau Claire, Wisconsin 54702, United States; Department of Kinesiology, McPhee Physical Education Center 221, University of Wisconsin Eau Claire, 105 Garfield Avenue, P.O. Box 4004, Eau Claire, Wisconsin 54702, United States; Department of Kinesiology, McPhee Physical Education Center 221, University of Wisconsin Eau Claire, 105 Garfield Avenue, P.O. Box 4004, Eau Claire, Wisconsin 54702, United States

**Keywords:** physical activity, metabolic syndrome risk, law enforcement, objective measurement

## Abstract

**Background:** There are limited data on objectively measured activity and postural behaviors of law enforcement officers (LEOs) in relation to risk of metabolic syndrome (MetS).

**Objectives:** To examine the associations between objectively measured activity and postural behaviors and MetS risk among LEOs.

**Methods**: Thirty-one LEOs, mean (SD) age 33 (10) years, participated in the study. LEOs had their metabolic risk factors measured using blood samples after fasting for at least 10 hours prior to testing. Participants wore activity-monitoring devices for 7 consecutive days during on-duty and off-duty shifts. Eighteen participants adhered to wearing the devices. Descriptive statistics were used to determine means for all MetS risk factors; time in intensity-specific physical activity behaviors; and time in various postural shifts. Correlation analyses were employed to examine relationships between activity behaviors, postures, and MetS risk factors.

**Results**: Over half (51.6%; *n* = 16) of the participants had 2 or more positive MetS risk factors. Mean (SD) on-duty sedentary behavior was 273 (59) minutes compared with off-duty sedentary behavior of 401 (146) minutes. Mean on-duty moderate-intensity activity was 236 (40) minutes compared with off-duty moderate-intensity activity of 305 (80) minutes. Average on-duty sitting time was 435 (69) minutes compared with off-duty sitting time of 528 (142) minutes. Average on-duty standing time was 116 (43) minutes compared with off-duty standing time of 171 (51) minutes. There were negative correlations between on-duty sedentary activity and Systolic Blood Pressure (*r* = −0.48) and Diastolic Blood Pressure (*r* = −0.48), respectively.

**Conclusions**: Law enforcement officers have unfavorable activity and postural behaviors during a typical day regardless of working status and may be at risk for developing MetS.

## Teaser

**Key points**

Law enforcement officers’ (LEOs’) occupational activities and postures vary throughout on-duty and off-duty times. Minimal data are available on off-duty behaviors of LEOs. The varied activities and postures can dramatically impact LEOs’ overall health, especially as it pertains to risk of metabolic syndrome (MetS). Previous studies that have examined LEOs’ activities and postures have relied heavily on self-reported data. MetS has been reported to have a higher prevalence in LEOs when compared with other occupations. The current study was the first to utilize objective measures to examine both activities and postures simultaneously in LEOs and their relationship to multiple MetS risk factors. LEOs achieved the recommended physical activity guidelines for moderate intensity (≥150–300 min) on both on-duty and off-duty days; however, these minutes were less than those spent in sedentary activity and sitting time regardless of working status. Despite meeting the recommended physical activity guidelines, more than half of the LEOs in the present study exhibited 2 or more of the risk factors for or had MetS. The current study demonstrated the feasibility of objectively measuring both physical activity and postural behaviors in LEOs during on-duty and off-duty shifts where limited research has been conducted previously. This study is the first to report objectively measured time spent in various postures among LEOs. The outcomes provide additional insights on the impact of LEOs’ occupational behaviors on metabolic risk factors and overall health.

## 1. Introduction

Law enforcement is generally seen as a sporadic profession where sedentary work can be interspersed with physically demanding occupational tasks (eg, civilian rescue, suspect pursuit, and restraint) and places great physiological stress on those in this occupation.^1^^,^^2^ The consequences may occur either during the individual’s professional career or become clinically evident only after retirement.^2^ Law enforcement officers are required to complete many fitness tasks with endurance, strength, and power, but most of their on-duty hours include occupational sitting (i.e., sitting in patrol cars, writing reports, and examining the criminal cases). This situation places law enforcement officers (LEOs) at a high risk of metabolic syndrome (MetS) and a decline in physical fitness level.^1^^,^^3^ Prospective observational evidence suggests that high volumes of sedentary behavior are associated with an elevated risk for all-cause mortality, cardiovascular disease incidence and mortality, type 2 diabetes incidence, and some cancers, particularly among those who are not achieving recommended amounts of moderate-to-vigorous-intensity physical activity.^3^^,^^4^ An accumulating body of acute experimental studies suggests that specific patterns of sedentary time (ie, whether sedentary behavior is undertaken in more prolonged or shorter bout durations) may be differentially associated with several cardiometabolic risk biomarkers and premature mortality.^4^

Previous studies have examined self-reported behavioral factors that influence obesity in LEOs and have determined that the differences in behaviors of those who were obese and non-obese were influenced by physical activity behavior.^5–7^ One previous study objectively measured MetS risk in LEOs and found at least 27% of research participants met the criteria for MetS.^5^ Most of the available data on activity and postural behaviors (ie, sitting and standing) of LEOs have been measured via self-report.^5–7^ There are limited data on objectively measured physical activity behaviors and no data on objectively measured postural behaviors of LEOs in the existing literature. It is also unclear from the available literature on LEOs if patterns of activity and postural behaviors change based on on-duty and off-duty work shifts.^3^^,^^8^ This information could provide insight on possible compensatory behaviors being made to offset the negative consequences of occupational tasks. Due to frequent bouts of on-duty sedentary behavior, this may place them at a greater risk for developing MetS later in their career. The purpose of this study was to examine the associations between objectively measured activity and postural behaviors and MetS risk among LEOs regardless of work shifts.

## 2. Methods

### 2.1. Subjects

Thirty-one (3 females and 28 males) active duty LEOs (mean [SD] age 33.1 [9.8] years) were recruited from local law enforcement agencies. Height, weight, and waist circumference were measured to the nearest 0.5 cm or 0.1 kg. Participant demographic data are provided in Table 1. Eighteen participants adhered to wearing the activity devices for the 7-day monitoring period. Prior to participation, written informed consent was provided in accordance with institutional review board policy and procedures.

**Table 1:** Descriptive statistics of participant characteristics and metabolic risk factors (*n* = 31).

	**Mean (SD)**
Age, years	33.1 (9.8)
Height, cm	173.1 (8.7)
Weight, kg	73.0 (12.1)
Body mass index, kg/m^2^	26.6 (4.2)
SBP, mmHg	124.9 (11.5)
DBP, mmHg	81.7 (8.7)
WC, cm	90.7 (11.6)
HDL, mg/dL	50.1 (20.2)
TGL, mg/dL	119.9 (81.5)
FBG, mg/dL	97.1 (9.1

Abbreviations: DBP, diastolic blood pressure; FBG, fasting blood glucose; HDL, high-density lipoprotein; LDL, low-density lipoprotein; SBP, systolic blood pressure; TC, total cholesterol; TGL, triglycerides;WC, waist circumference.

### 2.2. Physical activity and postural behaviors

The activPAL (PAL Technologies Ltd, Glasgow, UK) is a small device worn on the mid-anterior position of the thigh. Using the proprietary algorithm in the manufacturer-provided software, the final outputs for body postures (lying/sitting, standing, and stepping) are provided. The activPAL software version 7.1.18 was used to initialize the device and to download data.^9^ Law enforcement officers wore the activPal on the mid-thigh of the non-dominant leg. The ActiGraph wGT3X-BT accelerometer was also used in this study. This is a body-worn device that measures and records physical movement associated with daily activity and sleep.^10^^,^^11^ The ActiLife software version 6.11.9 was used to initialize the device and to download data. Law enforcement officers wore the ActiGraph on the non-dominant wrist.

### 2.3. Biometric measures

The basic anthropometric measurements that were taken included height, weight, waist circumference, and blood pressure. The same electronic scale (Seca 220) was used to measure height and weight to ensure consistency in measurements. Additionally, height was measured using the stadiometer attached to the scale. Waist circumference was measured using a Gulick tape measure, which consists of a tensiometer and a flexible plastic tape measure to guarantee equal tension on every waist circumference measurement, therefore ensuring intra-rater reliability.

Further, a standard sphygmomanometer and stethoscope were used to measure systolic and diastolic blood pressure (mmHg) on the right side in a seated position after 5 minutes of rest. The Cholestech LDX analyzer (Cholestech Corporation, Hayward, CA, USA) met current National Cholesterol Education Program guidelines for measuring total cholesterol. In addition, it was discovered that the high-density lipoprotein cholesterol measures were less accurate but that Cholestech was still a valid option for analyzing lipid profiles in non-medical settings.

**Table 2:** Results of paired samples *t* tests comparing physical activity and postural behaviors between on-duty and off-duty days (*n* = 18).^a^

	**On-duty shift only**	**On-duty day** ^**b**^	**Off-duty day**
**AG sedentary**	273 (59)	441 (110)	401 (146)
**AG light**	97 (20)	138 (33)	98 (26)^*^
**AG moderate**	236 (40)	370 (80)	305 (80)^**^
**AG vigorous**	0.6 (3)	3 (9.0)	3 (13)
**PAL sitting**	435 (69)	671 (148)	528 (142)^*^
**PAL standing**	116 (43)	193 (45)	171 (51)
**PAL stepping**	49 (18)	76 (24)	97 (36)

a Values are mean (SD) in minutes. ^*^Denotes significant difference between on-duty and off-duty days at *P* < .0071 (Bonferroni-adjusted α of .05/7); ^**^denotes significant difference between on-duty and off-duty days at *P* < .001.

b Values for On-duty day include on-duty shift time and on-duty day leisure time combined.

Abbreviations: AG, ActiGraph worn on non-dominant wrist; PAL, activPAL worn on mid-thigh of non-dominant leg.

### 2.4. Procedures

Law enforcement officers were asked to wear activity-monitoring devices for 7 consecutive days during on-duty and off-duty times while also maintaining an activity log. Participants wore an activPAL monitor placed on the non-dominant thigh that measured posture, and an ActiGraph worn on the non-dominant wrist that measured activity behaviors continuously over a 7-day measurement period, except during water-based activities. Data obtained on each device were downloaded and screened to ensure accurate recording. Participants were asked to fast for 10+ hours prior to visiting the laboratory for blood sample collection to measure metabolic risk factors using the Cholestec LDX. Measurements included waist circumference (cm), blood pressure (mmHg), Fasting Blood Glucose (mg/dL), Triglyceride level (mg/dL), High-Density Lipoprotein level (mg/dL), height (cm), and weight (kg).

### 2.5. Data analysis

All data were exported to Microsoft Excel and were separated into 24-hour time frames that began at the start of each participant’s scheduled shift. This was done to ensure consistent collection of activity for the entirety of the LEO’s shift in one 24-hour period. Each day was split into an on- or off-duty day. If the LEO was on-duty, the 24-hour period was then split into on-duty time (time spent working) and on-duty leisure time (time not working during that same day). If the officer was not working on a given day, the entire day was considered off-duty time. Captured activity intensities for each day were determined using metabolic equivalents (METs) from ActiGraph-determined values. Sedentary behavior was defined as ≤1.5 METs; light-intensity physical activity was defined as ≥1.6 to ≤2.9 METs; moderate-intensity physical activity was defined as ≥3.0 to ≤5.9 METs; and vigorous-intensity physical activity was defined as ≥6.1 METs.^13^

Descriptive statistics were used to determine means for all metabolic risk factors; to determine time spent in activity behaviors (sedentary, light, moderate, and vigorous); and to determine time spent in various postures (sitting, standing, and stepping) during on-duty and off-duty work shifts. Paired samples *t* tests, using a Bonferroni-adjusted α of .05/7 = .0071, were employed to examine the differences in physical activity behaviors and postural shifts between on-duty and off-duty days. Pearson correlation analyses (α = .05) were used to examine relationships between various physical activity behaviors, postural shifts, and metabolic risk factors. Spearman’s ρ non-parametric correlation analyses (α = .05) were employed to examine the correlations between a total number of positive metabolic risk factors (ordinal scale) and on-duty and off-duty physical activity behaviors. Statistical analyses were performed using IBM SPSS software version 29.0 (IBM Statistical Package for the Social Sciences, Inc., Chicago, IL, USA).

## 3. Results

Descriptive statistics for metabolic risk factors are provided in Table 1. Descriptive statistics and paired samples *t* test results for activity behaviors and changes in posture are provided in Table 2. Of a total of 31 participants, 16.1% (*n* = 5) had 3 or more positive MetS risk factors, and 35.5% (*n* = 11) had 2 or more positive MetS risk factors. Eighteen participants wore ActiGraph and activPAL devices over a 7-day period. A minimum of 4 on-duty days and 3 off-duty days were used in the study. On-duty shifts were typically 10 hours in duration. On-duty shifts were a mix of day and night shifts. Average (SD) on-duty sitting time was 435 (69) minutes compared with off-duty sitting time of 528 (142) minutes. Average on-duty standing time was 116 (43) minutes compared with off-duty standing time of 171 (51) minutes. Mean on-duty sedentary activity was 273 (59) minutes compared with off-duty sedentary activity of 401 (146) minutes. Mean on-duty moderate activity was 236 (40) minutes compared with off-duty moderate activity of 305 (80) minutes. There was a moderate negative correlation between on-duty sedentary activity and systolic blood pressure (*r* = −0.48, *P* < .05) and diastolic blood pressure (*r* = −0.48, *P* < .05), respectively. There was a positive correlation between on-duty stepping time and serum triglycerides (*r* = 0.51, *P* < .05), and a positive correlation between off-duty sitting and fasting blood glucose (*r* = 0.64, *P* < .01). There was a positive correlation between off-duty moderate-to-vigorous activity and waist circumference (*r* = 0.62, *P* < .01). Spearman’s ρ indicated a positive correlation between on-duty light-intensity physical activity and number of positive metabolic risk factors (ρ = 0.601, *P* = .008). There was also a positive correlation between on-duty day (combines on-duty shift time and on-duty day leisure time) light-intensity physical activity and number of positive metabolic risk factors (ρ = 0.645, *P*= .004).

## 4. Discussion

The purpose of this study was to examine the associations between objectively measured physical activity and postural behaviors with MetS risk among LEOs. According to the American Heart Association, the chance of developing cardiovascular disease is much higher when more than 1 risk factor for MetS is present. More than half of the LEOs in the present study exhibited 2 or more of the risk factors for MetS. The high prevalence of MetS risk observed in the current study is consistent with previous studies that have reported MetS prevalence percentages ranging from 26.7 to 36.4 among LEOs.^3^^,^^5^ The outcomes of this study also demonstrate that physical inactivity and obesity are associated with MetS risk among LEOs.^3^^,^^5^^,^^6^

The study results indicated that LEOs achieved the recommended physical activity guidelines for moderate intensity (≥150–300 min) on both on-duty and off-duty days; however, these minutes were less than those spent in sedentary activity and sitting time regardless of working status. Despite meeting the recommended physical activity guidelines, more than half of the LEOs in the present study exhibited 2 or more of the risk factors for or had MetS.

Utilizing the SenseWear Pro3 Armband, Ramey et al^8^ found that most LEOs (regardless of rank) engaged in sedentary and light-intensity physical activity during both on-duty and off-duty shifts. Our study results support these findings, demonstrating that more time was spent in these 2 physical activity categories when compared with moderate-to-vigorous physical activity regardless of working status. To our knowledge, this is the only other study that has objectively measured physical activity in LEOs and considered working status (on-duty vs off-duty). When comparing the results with self-reported physical activity behaviors of LEOs, it corroborates findings that this population does not engage in sufficient amounts of physical activity to offset deleterious health consequences regardless of working status.^5–7^ Clemes et al^14^ found that office workers accumulated significantly higher levels of sedentary behavior and lower levels of light-intensity physical activity on workdays in comparison with non-work days. When comparing our results with those found in office workers during workdays and non-work days, LEOs accumulated more sedentary time during off-duty shifts compared with their on-duty shifts with no differences in light-intensity physical activity.^14^ When considering the reported times spent in sedentary and light-intensity physical activity measured via accelerometry with the general US population, LEOs are at greater risk for experiencing stroke.^15^ It is recommended that behavioral modifications should be made to increase light-intensity physical activity while reducing the time spent in sedentary activity during both on-duty and off-duty shifts for LEOs.^12^

This study is the first to report objectively measured time spent in various postures among LEOs. Our results indicate that regardless of working status, this group spent more time sitting than either standing or stepping. The deleterious effects of long-term uninterrupted sitting have been well documented,^4^ and it appears that LEOs are vulnerable to these consequences. Our study results indicate that LEOs do not sufficiently compensate by increasing their physical activity or reducing their sitting time outside of work. It is recommended that behavioral modifications should be made through occupational interventions to reduce workplace and leisure-time sitting behaviors. Future studies should examine variables from activPAL daily bouts of consecutive sitting at <10 minutes, 10-60 minutes, 61-90 minutes, >90 minutes and metabolic risk factors in LEOs.

In a comprehensive review of cardiovascular disease risk factors among LEOs, Zimmerman^2^ identified both traditional risks, including hypertension, hyperlipidemia, MetS, cigarette smoking, overweight/obesity, and a sedentary lifestyle along with occupation-specific risk factors that include sudden physical exertion, acute and chronic psychological stress, shift work, and noise. Most of the available research to corroborate these findings was from self-report measures.^1^^,^^3^^,^^6^ High amounts of sedentary time (642 min/day) are associated with higher risk of mortality.^12^ Despite LEOs meeting the physical activity guidelines, their time spent in sedentary behavior (≥273 min on-duty and ≥401 min off-duty) and sitting time (≥435 min on-duty and ≥528 min off-duty) offset the benefits of time spent in moderate physical activity. The study findings provide additional objectively measured evidence for both identified traditional and occupational risk factors for cardiovascular disease among LEOs.

After accounting for body mass index (BMI) status, Anderson et al^5^ found that self-reported physical activity levels were no longer associated with MetS risk in LEOs. In contrast, after accounting for BMI status in our study, results indicated positive associations with waist circumference (*r* = 0.93, *P* < .05) and triglycerides (*r* = 0.53, *P* < .05) MetS risk factors. Future studies should consider including measures of nutritional intake along with objective measures of physical activity, postural shifts, and MetS to capture a more holistic health risk profile of LEOs. Limitations of the study include the exclusion of information pertaining to LEOs’ department type, rank, and years of service, which have all been shown to impact activity level and health status.^8^

## 5. Conclusion

The current study demonstrated the feasibility of objectively measuring both physical activity and postural behaviors in LEOs during on-duty and off-duty shifts where limited research has been conducted previously. Law enforcement officers may be at risk of developing MetS and have unfavorable activity and postural behaviors during a typical day regardless of working status. Law enforcement officers’ activity behaviors and postural shifts did not vary much during on-duty and off-duty times. The study outcomes support the susceptibility of LEOs to cardiovascular disease risk^2^^,^^3^ and the development of obesity^1^^,^^6^ due to both on-duty and off-duty occupational behaviors. Inclusion of the objective measurements of daily activity behaviors and changes in posture accumulated in LEOs provide additional insights into the impact of occupational behaviors on metabolic risk factors and overall health.

## Author contributions

M.J., S.B., M.H., K.S., C.B., A.B., and J.J. met the ICMJE definition of authorship.

## Funding

None declared.

## Conflicts of interest

The authors report no conflicts of interest.

## Data availability

The data that support the findings of this study are available on request from the corresponding author. The data are not publicly available due to privacy requirements or ethical restrictions.
